# Crystal structure and Hirshfeld surface analysis of dimethyl (1R*,3aS*,3a^1^ R*,6aS*,9R*,9aS*)-3a^1^,5,6,9a-tetra­hydro-1*H*,4*H*,9*H*-1,3a:6a,9-di­epoxy­phenalene-2,3-di­carboxyl­ate

**DOI:** 10.1107/S2056989019003499

**Published:** 2019-03-20

**Authors:** Kseniia A. Alekseeva, Pavel V. Raspertov, Sevim Türktekin Çelikesir, Mehmet Akkurt, Flavien A. A. Toze, Elena A. Sorokina

**Affiliations:** a Organic Chemistry Department, Faculty of Science, Peoples’ Friendship University of Russia (RUDN University), 6 Miklukho-Maklaya St., Moscow 117198, Russian Federation; b Department of Physics, Faculty of Sciences, Erciyes University, 38039 Kayseri, Turkey; c Department of Chemistry, Faculty of Sciences, University of Douala, PO Box 24157, Douala, Republic of Cameroon

**Keywords:** crystal structure, di­epoxy­phenalene, fused hexa­cyclic system, C—H⋯O hydrogen bonds, Hirshfeld surface analysis

## Abstract

The title di­epoxy­phenalene derivative, C_17_H_18_O_6_, comprises a fused cyclic system containing four five-membered rings (two di­hydro­furan and two tetra­hydro­furan) and one six-membered ring (cyclo­hexa­ne). The five-membered di­hydro­furan and tetra­hydro­furan rings adopt envelope conformations, and the six-membered cyclo­hexane ring adopts a distorted chair conformation. Two methyl carboxyl­ate groups occupy adjacent positions (2- and 3-) on a tetra­hydro­furan ring. In the crystal, two pairs of C—H⋯O hydrogen bonds link the mol­ecules to form inversion dimers, enclosing two *R* ^2^_2_(6) ring motifs, that stack along the *a*-axis direction and are arranged in layers parallel to the *bc* plane.

## Abstract 2

In the title di­epoxy­phenalene derivative, two di­hydro­furan and two tetra­hydro­furan rings, as well as one cyclo­hexane ring, are fused together with two methyl carboxyl­ate groups in positions 2- and 3-. In the crystal, two pairs of C—H⋯O hydrogen bonds link the mol­ecules to form inversion dimers, enclosing two 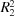
(6) ring motifs.

## Chemical context   

Reactions totally depending on thermodynamic and kinetic control are infrequently found in the field of organic synthesis, at the same time such transformations are very perspective and attractive from a practical point of view since they allow the direction of the reaction to be changed radically by varying only one of the reaction parameters (usually the catalyst or temperature).

The first example of kinetic/thermodynamic control in the course of the Diels–Alder reaction was reported in 1948 (Woodward & Baer, 1948 ▸). Since then, the reversibility of the [4 + 2] cyclo­addition was observed many times for examples of a broad range of dienes and dienophiles, including alkynes and furans (Boutelle & Northrop, 2011 ▸; Taffin *et al.*, 2010 ▸; White *et al.*, 2000 ▸; Marchand *et al.*, 1998 ▸; Manoharan & Venuvanalingam, 1997 ▸; Bott *et al.*, 1996 ▸; Bartlett & Wu, 1985 ▸). From this diversity of diene/dienophile combinations, tandem and domino reactions of the [4 + 2] cyclo­addition based on acetyl­enic dienophiles are more inter­esting for the total synthesis of natural or bioactive products (Sears & Boger, 2016 ▸; Parvatkar *et al.*, 2014 ▸; Winkler, 1996 ▸). However, the range of bis-dienes suitable for such tandem transformations is very limited and, currently, there are only a few published examples of full kinetic/thermodynamic control in the course of the tandem intra­molecular [4 + 2] cyclo­addition (reactions leading to either kinetically or thermodynamically controlled products, depending on temperature; Marchionni *et al.*, 1996 ▸; Oh *et al.*, 2010 ▸; Criado *et al.*, 2010 ▸; Paquette *et al.*, 1978 ▸; Visnick & Battiste, 1985 ▸).

The present paper describes the uncommon thermal rearrangement of the ‘*pincer-adduct*’ (**1**) into the ‘*domino-adduct*’ (**2**) [the terminology and the mechanism of the reaction are given in references Borisova, Nikitina *et al.* (2018 ▸) and Borisova, Kvyatkovskaya *et al.* (2018 ▸); for references of works related to the present paper, see also Lautens & Fillion (1998 ▸), Lautens & Fillion (1997 ▸) and Domingo *et al.* (2000 ▸)]. The transformation proceeds through the reversible retro-Diels–Alder reaction of the kinetically controlled ‘*pincer-adduct*’ (**1**), followed by the repeated intra­molecular [4 + 2] cyclo­addition in an inter­mediate, leading to the formation of the thermodynamically controlled ’*domino-adduct*’ (**2**) in an almost qu­anti­tative yield.
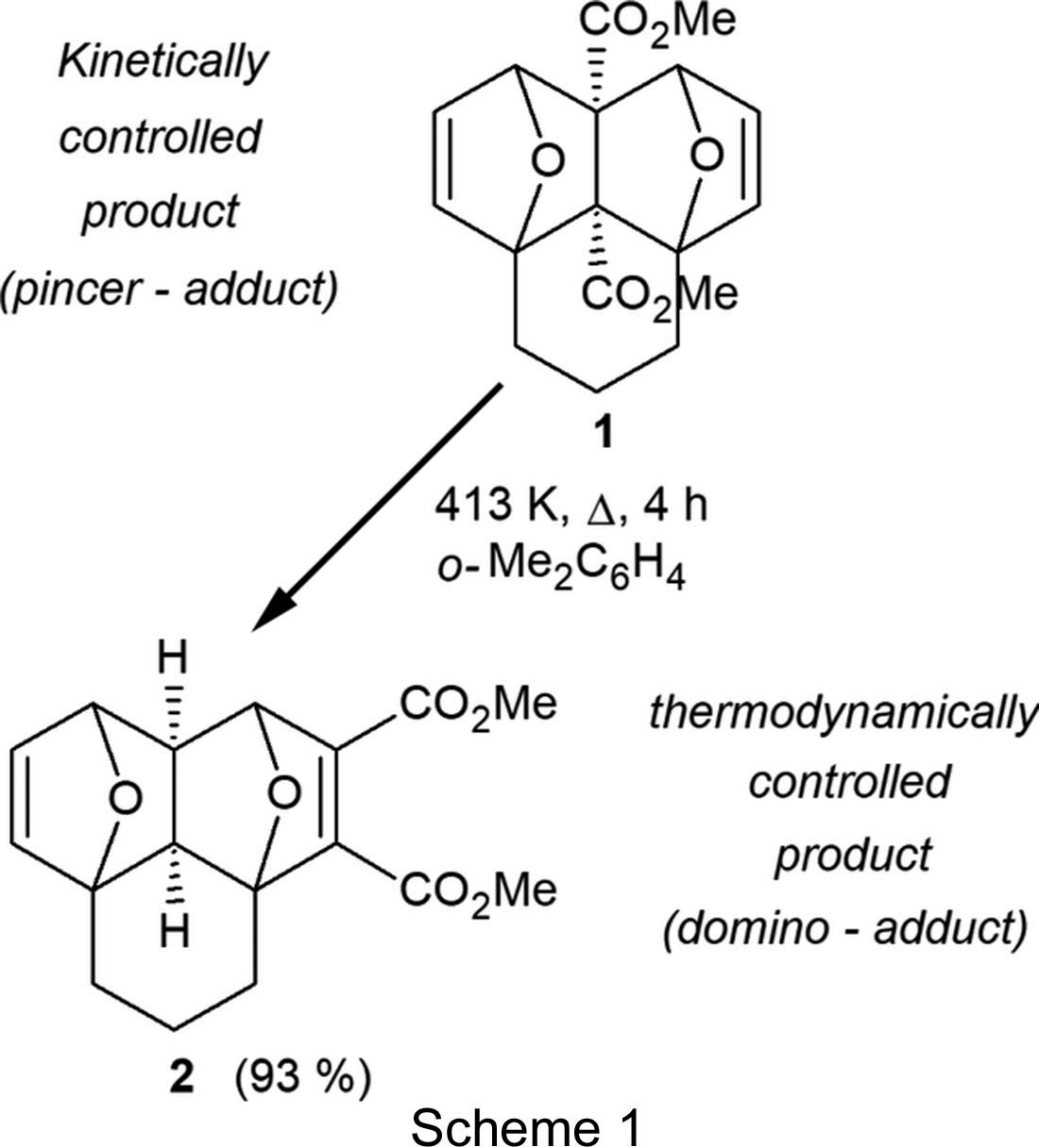


## Structural commentary   

**Figure 1:**
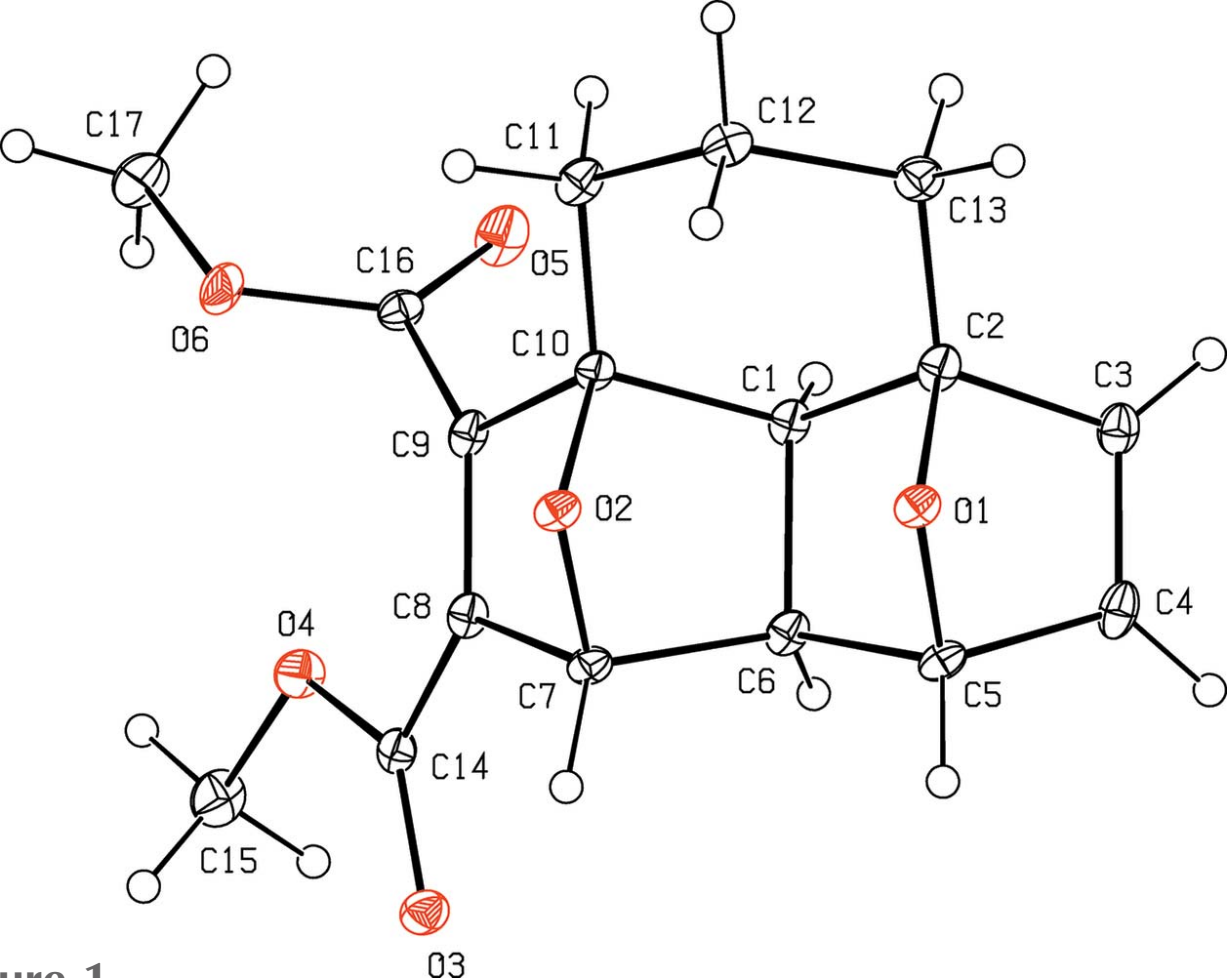
The mol­ecular structure of compound (**2**), with the atom labelling. Displacement ellipsoids are drawn at the 30% probability level.

The mol­ecule structure of compound (**2**) is illustrated in Fig. 1 ▸. It is made up from a fused cyclic system containing four five-membered rings (two di­hydro­furan and two tetra­hydro­furan) in the usual envelope conformations and a six-membered cyclo­hexane ring in a distorted chair conformation. The puckering parameters of the five-membered di­hydro­furan (A = O1/C1/C2/C5/C6 and B = O2/C1/C6/C7/C10) and tetra­hydro­furan (C = O1/C2–C5 and D = O2/C7–C10) rings are *Q*(2) = 0.5230 (18) Å and φ(2) = 178.1 (2)° for ring A, *Q*(2) = 0.5492 (17) Å and φ(2) = 182.3 (2)° for B, *Q*(2) = 0.5230 (18) Å and φ(2) = 1.0 (2)° for C, and *Q*(2) = 0.5303 (17) Å and φ(2) = 358.9 (2)° for D. The puckering parameters of the six-membered cyclo­hexane ring (C1/C2/C10–C13) are *Q*_T_ = 0.518 (2) Å, θ = 6.9 (2)° and φ = 178.2 (18)°. In positions 2- and 3-, *i.e.* on atoms C8 and C9 (Fig. 1 ▸), there are methyl carboxyl­ate substituents whose mean planes are inclined to the mean plane through atoms C7–C10 by 7.38 (13) and 70.65 (14)° for groups O3/O4/C14/C15 and O5/O6/C16/C17, respectively.

## Supra­molecular features and Hirshfeld surface analysis   

**Figure 2:**
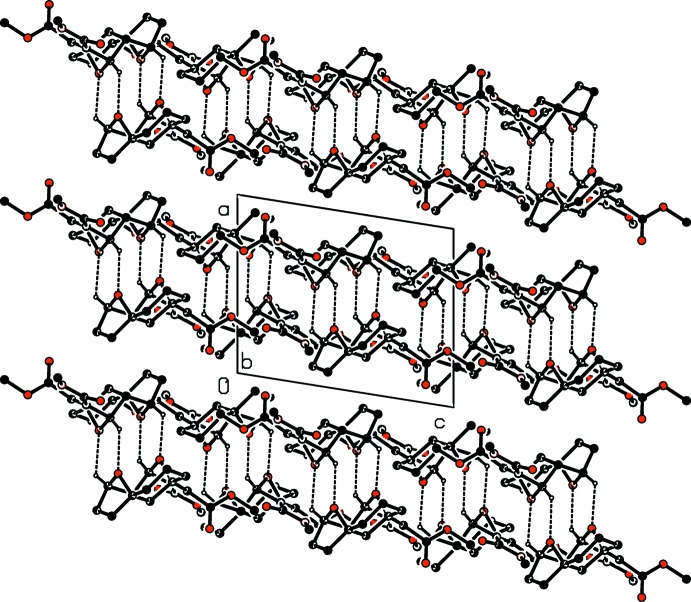
A viewed along the *b* axis of the crystal packing of compound (**2**), emphasizing the formation of C—H⋯O hydrogen-bonded dimers. Hydrogen bonds are shown as dashed lines (Table 1 ▸).

In the crystal, two pairs of C—H⋯O hydrogen bonds link the mol­ecules forming inversion dimers, enclosing two 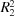
(6) ring motifs. The dimers stack along the *a*-axis direction and are arranged in layers parallel to the *bc* plane (Table 1 ▸ and Fig. 2 ▸). C—H⋯π and π–π inter­actions are not observed, but H⋯H contacts (Tables 2 ▸ and 3 ▸) dominate in the packing, as detailed in the next section.

## Hirshfeld surface analysis and two-dimensional fingerprint plots   

**Figure 3:**
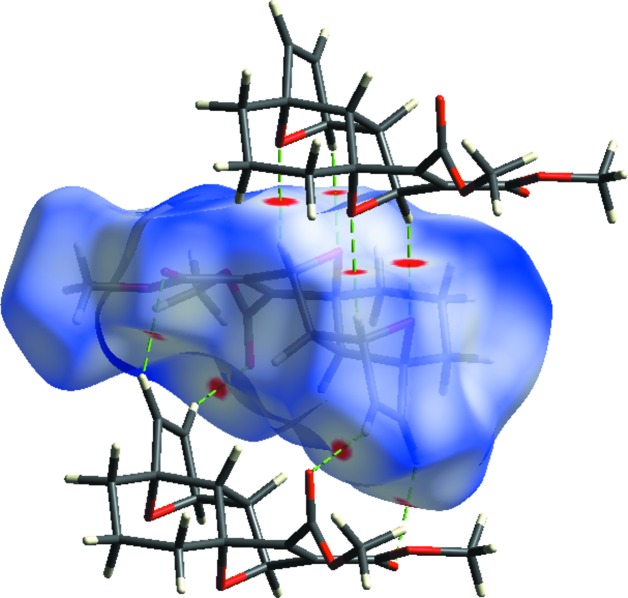
Hirshfeld surface of compound (**2**) mapped over *d*_norm_.

Hirshfeld surface and fingerprint plots were generated using *CrystalExplorer* (McKinnon *et al.*, 2007 ▸). Hirshfeld surfaces enable the visualization of inter­molecular inter­actions by different colours and colour intensity, representing short or long contacts and indicating the relative strength of the inter­actions. Fig. 3 ▸ shows the Hirshfeld surface of the title compound mapped over *d*_norm_, where it is evident from the bright-red spots appearing near the O atoms that these atoms play a significant role in the mol­ecular packing. The red spots represent closer contacts and negative *d*_norm_ values on the surface, corresponding to the C—H⋯O inter­actions.

**Figure 4:**
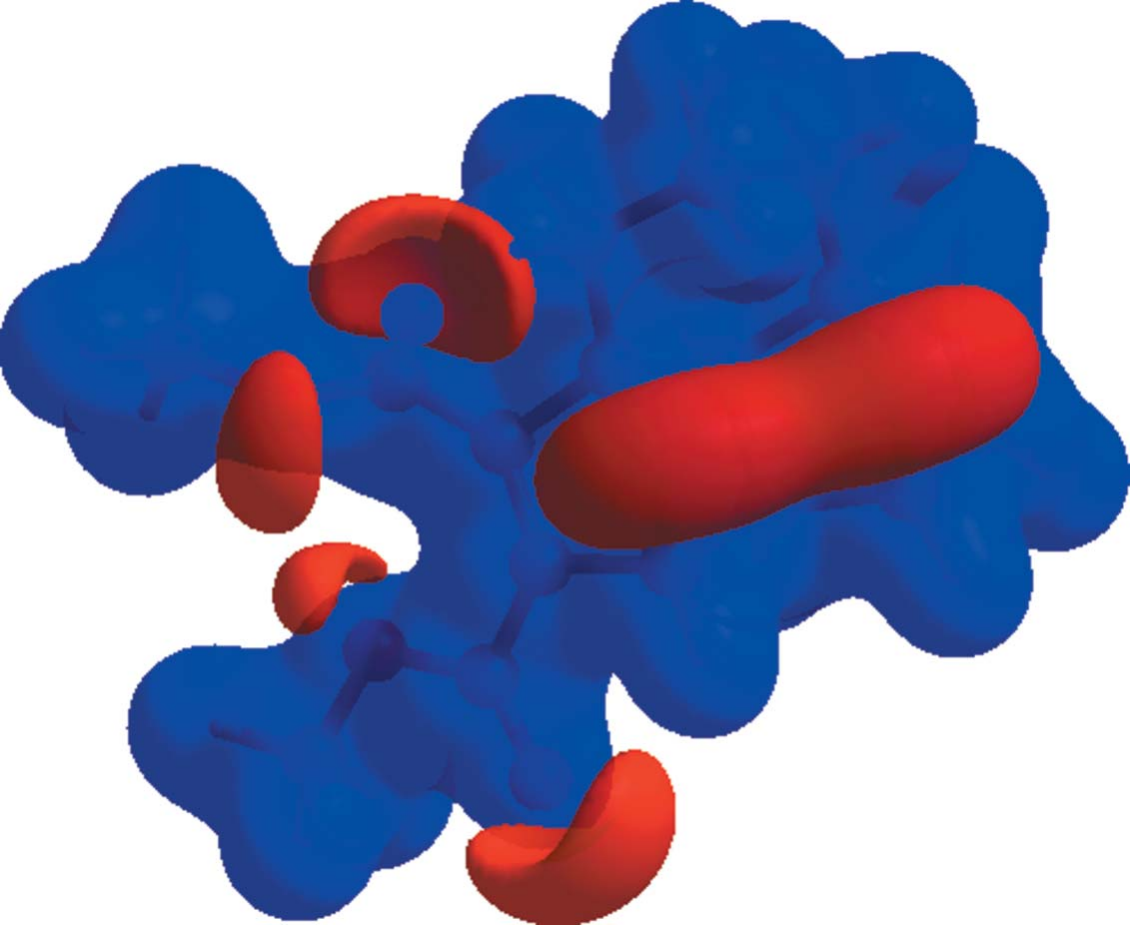
View of the three-dimensional Hirshfeld surface of compound (**2**) plotted over electrostatic potential energy in the range −0.0500 to 0.0500 a.u. using the STO-3 G basis set at the Hartree–Fock level of theory. Hydrogen-bond donors and acceptors are shown as blue and red regions around the atoms corresponding to positive and negative potentials, respectively.

**Figure 5:**
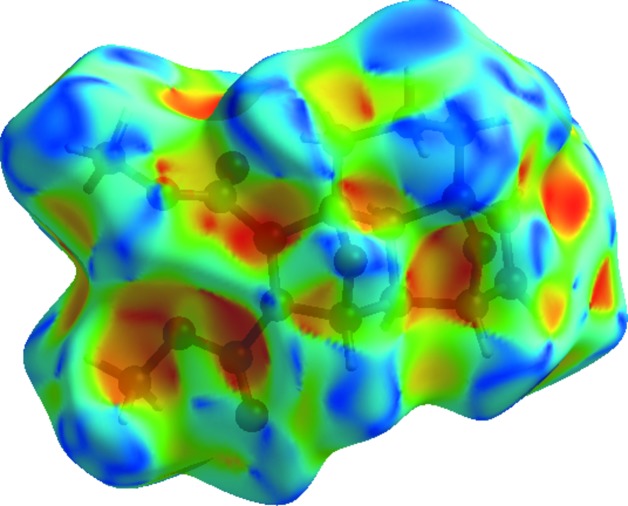
Hirshfeld surface of compound (**2**) plotted over shape index.

The bright-red spots indicate their roles as the respective donors and/or acceptors; they also appear as blue and red regions corresponding to positive and negative potentials on the Hirshfeld surface mapped over electrostatic potential (Fig. 4 ▸; Spackman *et al.*, 2008 ▸; Jayatilaka *et al.*, 2005 ▸). The blue regions indicate the positive electrostatic potential (hydrogen-bond donors), while the red regions indicate the negative electrostatic potential (hydrogen-bond acceptors). The shape index of the Hirshfeld surface is a tool to visualize the π–π stacking by the presence of adjacent red and blue triangles; if there are no adjacent red and/or blue triangles, then there are no π–π inter­actions. Fig. 5 ▸ clearly suggest that no π–π inter­actions are present in the title compound.

**Figure 6:**
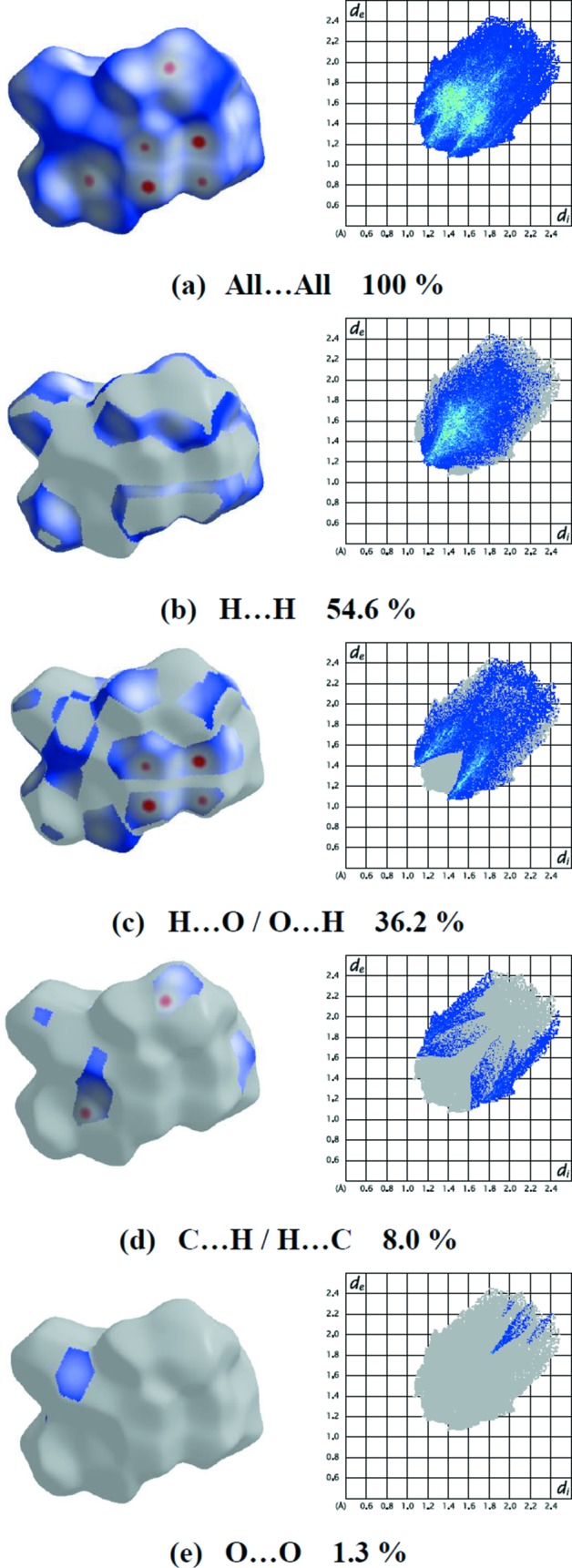
The 2D fingerprint plots of compound (**2**), showing (*a*) all inter­actions, and delineated into (*b*) H⋯H, (*c*) O⋯H/H⋯O, (*d*) C⋯H/H⋯C and (*e*) O⋯O inter­actions [*d*_e_ and *d*_i_ represent the distances from a point on the Hirshfeld surface to the nearest atoms outside (external) and inside (inter­nal) the surface, respectively].

The percentage contributions of various contacts to the total Hirshfeld surface are given in Table 3 ▸ and are also shown as two-dimensional (2D) fingerprint plots in Fig. 6 ▸. The H⋯H inter­actions appear in the middle of the scattered points in the 2D fingerprint plots with an overall contribution to the Hirshfeld surface of 54.6% (Fig. 6 ▸ *b*). The contribution from the O⋯H/H⋯O contacts, corresponding to C—H⋯O inter­actions, is represented by a pair of sharp spikes characteristic of a strong hydrogen-bonding inter­action (36.2%, Fig. 6 ▸ *c* and Tables 2 ▸ and 3 ▸). The small percentage contributions from the remaining inter­atomic contacts are summarized in Table 3 ▸ and indicated by their fingerprint plots for C⋯H/H⋯C (Fig. 6 ▸ *d*) and O⋯O (Fig. 6 ▸ *e*). The large number of H⋯H and O⋯H/H⋯O inter­actions suggest that van der Waals inter­actions and hydrogen bonding play the major roles in the crystal packing (Hathwar *et al.*, 2015 ▸).

## Database survey   

A search of the Cambridge Structural Database (CSD, Version 5.40, February 2019; Groom *et al.*, 2016 ▸) for the di­epoxy­phenalene skeleton gave only 2 hits, *viz*. 9b-acetyl-9a-meth­oxy­carbonyl-1,3a:6a,9-diep­oxy-4,5,6,9-tetra­hydro­phena­lene (CSD refcode RUSGOB; Lautens & Fillion, 1997 ▸) and 9a-benzene­sulfonyl-1,3a:6a,9-diep­oxy-9b-meth­oxy­carbonyl-4,5,6,9-tetra­hydro­phenalene (RUSHAO; Lautens & Fillion, 1997 ▸). A search for the di­epoxy­benzo[*de*]iso­quinoline skelton gave 8 hits, three of which are very similar to compounds (**1**) and (**2**), *viz*. 2-benzyl-6a,9b-bis­(tri­fluoro­meth­yl)-2,3,6a,9b-tetra­hydro-1*H*,6*H*,7*H*-3a,6:7,9a-di­epoxy­benzo[*de*]iso­quinoline (CSD refcode HENLAQ; Borisova, Nikitina *et al.*, 2018 ▸), 2-benzyl-4,5-bis­(tri­fluoro­meth­yl)-2,3,6a,9b-tetra­hydro-1*H*,6*H*,7*H*-3a,6:7,9a-di­epoxy­benzo[*de*]iso­quinoline (HENLEU; Borisova, Nikitina *et al.*, 2018 ▸) and dimethyl (3a*S*,6*R*,6a*S*,7*S*)-2-(2,2,2-tri­fluoro­acet­yl)-2,3-di­hydro-1*H*,6*H*,7*H*-3a,6:7,9a-di­epoxy­benzo[*de*]iso­quinoline-3a^1^,6a-di­carboxyl­ate (LIRKAB; Atioğlu *et al.*, 2018 ▸).

In the crystal of HENLAQ, inversion-related mol­ecules are linked into dimers by pairs of C—H⋯O hydrogen bonds, and the dimers lie in layers parallel to (100). C—H⋯π inter­actions are also observed, together with intra­molecular F⋯F contacts. The asymmetric unit of HENLEU contains two independent mol­ecules. In the crystal, mol­ecules are linked by C—H⋯O and C—H⋯F hydrogen bonds, forming columns along [010]. Likewise, C—H⋯π inter­actions and F⋯F intra­molecular contacts are also present. In the crystal structure of LIRKAB, inter­molecular C—H⋯O inter­actions involving the O atoms of the carbonyl groups, the oxygen bridgehead atoms and the meth­oxy O atoms, as well as C—H⋯F hydrogen bonds, define the crystal packing. These packing features lead to the formation of a supra­molecular three-dimensional structure. C—H⋯π and π–π inter­actions are not observed, but H⋯H inter­actions dominate in the packing. This situation is similar to that in the crystal of the title compound.

## Synthesis and crystallization   

The synthesis of the title compound (**2**) is illustrated in the Scheme. Compound (**1**) (0.89 g, 2.81 mmol) was dissolved in dry *o*-Me_2_C_6_H_4_ (15 ml) and then heated under reflux for 4 h at ∼413 K (thin-layer chromatography monitoring). The reaction mixture was cooled and the solvent removed under reduced pressure. The residue was purified by recrystallization from an EtOAc/hexane mixture (1:1 *v*/*v*) to give compound (**2**) as large colourless prismatic crystals [0.82 g, 2.61 mmol, 93%; m.p. 410.4–411.8 K (hexa­ne/EtOAc)]. ^1^H NMR (400 MHz, CDCl_3_): δ 6.43 (1H, *dd*, *J* = 1.8 and *J* = 5.6 Hz, H-8), 6.27 (1H, *d*, *J* = 5.6 Hz, H-9), 5.09 (1H, *s*, H-1), 4.88 (1H, *d*, *J* = 1.8 Hz, H-9), 3.78 (3H, *s*, CO_2_Me), 3.73 (3H, *s*, CO_2_Me), 2.23–2.17 (3H, *m*, H-4A, H-6A and H-9a), 2.00–1.88 (4H, *m*, H-4B, H-6B, H-5A and H-9b) 1.71–1.68 (1H, *m*, H-5B). ^13^C NMR (100 MHz, CDCl_3_): δ 164.7 (*C*O_2_Me), 162.6 (*C*O_2_Me), 150.6 (C-3), 143.8 (C-2), 140.8 (C-7), 138.5 (C-8), 89.3 (C-3a), 85.8 (C-6a), 81.3 (C-1), 80.5 (C-9), 52.2 (C-9a), 52.0 (2 × CO_2_ *Me*), 49.8 (C-9b), 26.7 (C-9), 25.0 (C-6), 17.2 (C-5). IR ν_max_/cm^−1^ (KBr): 1709, 1628, 1284, 1261. HRMS (ESI–TOF): calculated for C_17_H_18_O_6_ [*M* + H]^+^ 318.1103; found 318.1125.

## Refinement   

Crystal data, data collection and structure refinement details are summarized in Table 4 ▸. All H atoms were fixed and allowed to ride on the parent atoms, with C—H = 0.95–1.00 Å, and with *U*_iso_(H) = 1.5*U*_eq_(C) for methyl H atoms and 1.2*U*_eq_(C) for other H atoms.

## Supplementary Material

Crystal structure: contains datablock(s) 2, Global. DOI: 10.1107/S2056989019003499/rz5253sup1.cif

CCDC reference: 1902671

Additional supporting information:  crystallographic information; 3D view; checkCIF report

## Figures and Tables

**Table 1:** Hydrogen-bond geometry (Å, °)

*D*—H⋯*A*	*D*—H	H⋯*A*	*D*⋯*A*	*D*—H⋯*A*
C5—H5⋯O2^i^	1.00	2.58	3.330 (2)	132
C7—H7⋯O1^i^	1.00	2.52	3.351 (2)	140

Symmetry code: (i) 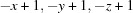.

**Table 2:** Summary of short inter­atomic contacts (Å) in the crystal of compound (2).

Contact	Distance	Symmetry operation
H7⋯O1	2.52	1 − *x*, 1 − *y*, 1 − *z*
H13*B*⋯H17*B*	2.49	*x*, 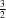 − *y*, − 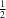 + *z*
H15*C*⋯H12*A*	2.53	1 − *x*, − 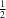 + *y*, 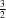 − *z*
H15*A*⋯H3	2.56	−*x*, 1 − *y*, 1 − *z*
H6⋯H15*B*	2.57	*x*, 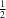 − *y*, − 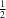 + *z*
H17*A*⋯O5	2.90	−*x*, 1 − *y*, 2 − *z*
H15*B*⋯H6	2.57	*x*, 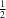 − *y*, 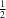 + *z*
H5⋯H17*C*	2.48	*x*, *y*, −1 + *z*

**Table 3:** Percentage contributions of inter­atomic contacts to the Hirshfeld surface of compound (2)

Contact	Percentage contribution
H⋯H	54.6
O⋯H/H⋯O	36.2
C⋯H/H⋯C	8.0
O⋯O	1.1

**Table 4:** Experimental details

Crystal data	
Chemical formula	C_17_H_18_O_6_
*M*_r_	318.31
Crystal system, space group	Monoclinic, *P*2_1_/*c*
Temperature (K)	100
*a*, *b*, *c* (Å)	9.3903 (19), 14.157 (3), 11.520 (2)
β (°)	99.032 (3)
*V* (Å^3^)	1512.5 (5)
*Z*	4
Radiation type	Synchrotron, λ = 0.96990 Å
μ (mm^−1^)	0.23
Crystal size (mm)	0.35 × 0.15 × 0.10

Data collection	
Diffractometer	MAR CCD
Absorption correction	Multi-scan (*SCALA*; Evans, 2006 ▸)
*T*_min_, *T*_max_	0.918, 0.975
No. of measured, independent and observed [*I* > 2σ(*I*)] reflections	17699, 3216, 2464
*R*_int_	0.151
(sin θ/λ)_max_ (Å^−1^)	0.641

Refinement	
*R*[*F* ^2^ > 2σ(*F* ^2^)], *wR*(*F* ^2^), *S*	0.067, 0.192, 1.11
No. of reflections	3216
No. of parameters	211
H-atom treatment	H-atom parameters constrained
Δρ_max_, Δρ_min_ (e Å^−3^)	0.50, −0.41

Computer programs: *Automar* (Doyle, 2011 ▸), *iMosflm* (Battye *et al.*, 2011 ▸), *SHELXS97* (Sheldrick, 2008 ▸), *SHELXL2018* (Sheldrick, 2015 ▸), *ORTEP-3 for Windows* (Farrugia, 2012 ▸) and *PLATON* (Spek, 2009 ▸).

## supplementary crystallographic information

### Crystal data

**Table:**

C_17_H_18_O_6_	*F*(000) = 672
*M**_r_* = 318.31	*D*_x_ = 1.398 Mg m^−^^3^
Monoclinic, *P*2_1_/*c*	Synchrotron radiation, λ = 0.96990 Å
*a* = 9.3903 (19) Å	Cell parameters from 500 reflections
*b* = 14.157 (3) Å	θ = 3.5–35.0°
*c* = 11.520 (2) Å	µ = 0.23 mm^−^^1^
β = 99.032 (3)°	*T* = 100 K
*V* = 1512.5 (5) Å^3^	Prism, colourless
*Z* = 4	0.35 × 0.15 × 0.10 mm

### Data collection

**Table:**

MAR CCD diffractometer	2464 reflections with *I* > 2σ(*I*)
/f scan	*R*_int_ = 0.151
Absorption correction: multi-scan (*Scala*; Evans, 2006)	θ_max_ = 38.5°, θ_min_ = 3.6°
*T*_min_ = 0.918, *T*_max_ = 0.975	*h* = −11→12
17699 measured reflections	*k* = −17→14
3216 independent reflections	*l* = −14→14

### Refinement

**Table:**

Refinement on *F*^2^	Hydrogen site location: inferred from neighbouring sites
Least-squares matrix: full	H-atom parameters constrained
*R*[*F*^2^ > 2σ(*F*^2^)] = 0.067	*w* = 1/[σ^2^(*F*_o_^2^) + (0.0746*P*)^2^] where *P* = (*F*_o_^2^ + 2*F*_c_^2^)/3
*wR*(*F*^2^) = 0.192	(Δ/σ)_max_ < 0.001
*S* = 1.11	Δρ_max_ = 0.50 e Å^−^^3^
3216 reflections	Δρ_min_ = −0.40 e Å^−^^3^
211 parameters	Extinction correction: SHELXL2018 (Sheldrick, 2015), Fc^*^=kFc[1+0.001xFc^2^λ^3^/sin(2θ)]^-1/4^
0 restraints	Extinction coefficient: 0.038 (4)

### Special details

**Table:**

Geometry. All esds (except the esd in the dihedral angle between two l.s. planes) are estimated using the full covariance matrix. The cell esds are taken into account individually in the estimation of esds in distances, angles and torsion angles; correlations between esds in cell parameters are only used when they are defined by crystal symmetry. An approximate (isotropic) treatment of cell esds is used for estimating esds involving l.s. planes.

### Fractional atomic coordinates and isotropic or equivalent isotropic displacement parameters (Å^2^)

**Table:**

	*x*	*y*	*z*	*U*_iso_*/*U*_eq_	
C1	0.20277 (19)	0.59385 (13)	0.59498 (15)	0.0155 (5)	
H1	0.106665	0.591973	0.621979	0.019*	
C2	0.21999 (18)	0.67977 (14)	0.51388 (16)	0.0177 (5)	
C3	0.0861 (2)	0.67729 (14)	0.41833 (16)	0.0195 (5)	
H3	0.006228	0.719276	0.409799	0.023*	
C4	0.1054 (2)	0.60374 (14)	0.34962 (16)	0.0214 (5)	
H4	0.041452	0.582144	0.282721	0.026*	
C5	0.25131 (19)	0.56171 (14)	0.40095 (15)	0.0173 (5)	
H5	0.299224	0.523974	0.344653	0.021*	
C6	0.22826 (19)	0.50832 (14)	0.51569 (15)	0.0163 (5)	
H6	0.144221	0.464107	0.502585	0.020*	
C7	0.36433 (19)	0.46304 (13)	0.58988 (15)	0.0157 (5)	
H7	0.419362	0.418266	0.546483	0.019*	
C8	0.31304 (18)	0.42263 (13)	0.69973 (15)	0.0156 (5)	
C9	0.29437 (19)	0.49717 (13)	0.76792 (16)	0.0160 (5)	
C10	0.33216 (18)	0.58488 (13)	0.69840 (15)	0.0141 (5)	
C11	0.3755 (2)	0.67670 (13)	0.76102 (16)	0.0194 (5)	
H11A	0.465992	0.667147	0.816594	0.023*	
H11B	0.299650	0.695990	0.806996	0.023*	
C12	0.3976 (2)	0.75571 (15)	0.67394 (17)	0.0198 (5)	
H12A	0.481612	0.739906	0.635317	0.024*	
H12B	0.419398	0.815491	0.717708	0.024*	
C13	0.2644 (2)	0.77010 (14)	0.57926 (17)	0.0202 (5)	
H13A	0.183317	0.793440	0.616846	0.024*	
H13B	0.286127	0.818677	0.522740	0.024*	
C14	0.27146 (19)	0.32255 (14)	0.71005 (16)	0.0159 (5)	
C15	0.1868 (2)	0.20708 (15)	0.83169 (18)	0.0268 (5)	
H15A	0.104113	0.188440	0.773336	0.040*	
H15B	0.162026	0.200255	0.910798	0.040*	
H15C	0.269400	0.166559	0.823982	0.040*	
C16	0.22323 (19)	0.50325 (14)	0.87480 (16)	0.0163 (5)	
C17	0.2451 (3)	0.46966 (17)	1.07828 (17)	0.0322 (6)	
H17A	0.179303	0.415658	1.075185	0.048*	
H17B	0.191531	0.528174	1.085776	0.048*	
H17C	0.321654	0.463002	1.146134	0.048*	
O1	0.32913 (13)	0.64562 (9)	0.44759 (10)	0.0165 (4)	
O2	0.44470 (13)	0.54506 (9)	0.64055 (10)	0.0160 (4)	
O3	0.27815 (14)	0.26386 (10)	0.63425 (11)	0.0211 (4)	
O4	0.22345 (14)	0.30455 (10)	0.81243 (11)	0.0216 (4)	
O5	0.10290 (15)	0.53573 (11)	0.87145 (11)	0.0287 (4)	
O6	0.30886 (14)	0.47284 (10)	0.97057 (11)	0.0231 (4)	

### Atomic displacement parameters (Å^2^)

**Table:**

	*U*^11^	*U*^22^	*U*^33^	*U*^12^	*U*^13^	*U*^23^
C1	0.0115 (9)	0.0231 (12)	0.0132 (9)	0.0005 (7)	0.0054 (7)	0.0011 (8)
C2	0.0127 (9)	0.0273 (12)	0.0140 (9)	0.0018 (8)	0.0053 (7)	0.0002 (8)
C3	0.0177 (10)	0.0258 (12)	0.0155 (9)	0.0009 (8)	0.0043 (7)	0.0040 (8)
C4	0.0182 (10)	0.0328 (13)	0.0131 (9)	−0.0008 (8)	0.0028 (7)	0.0055 (8)
C5	0.0180 (10)	0.0214 (11)	0.0133 (9)	−0.0026 (8)	0.0051 (7)	−0.0039 (8)
C6	0.0135 (9)	0.0236 (12)	0.0129 (9)	−0.0010 (8)	0.0054 (7)	−0.0002 (8)
C7	0.0148 (9)	0.0193 (11)	0.0140 (9)	0.0003 (7)	0.0057 (7)	−0.0032 (8)
C8	0.0126 (9)	0.0216 (12)	0.0134 (9)	0.0003 (8)	0.0047 (7)	0.0015 (8)
C9	0.0129 (9)	0.0236 (12)	0.0120 (9)	−0.0001 (8)	0.0037 (7)	0.0020 (8)
C10	0.0125 (9)	0.0199 (11)	0.0112 (9)	0.0025 (7)	0.0055 (7)	−0.0007 (7)
C11	0.0164 (10)	0.0270 (12)	0.0156 (9)	−0.0019 (8)	0.0055 (7)	−0.0015 (8)
C12	0.0195 (10)	0.0208 (12)	0.0197 (10)	−0.0042 (8)	0.0051 (7)	−0.0025 (8)
C13	0.0213 (10)	0.0221 (12)	0.0186 (10)	0.0012 (8)	0.0072 (7)	−0.0008 (8)
C14	0.0119 (9)	0.0220 (12)	0.0143 (9)	0.0026 (7)	0.0032 (7)	0.0010 (8)
C15	0.0328 (12)	0.0273 (13)	0.0219 (10)	−0.0056 (10)	0.0088 (9)	0.0049 (9)
C16	0.0183 (10)	0.0167 (11)	0.0146 (9)	−0.0027 (7)	0.0048 (7)	−0.0013 (7)
C17	0.0488 (15)	0.0368 (14)	0.0141 (10)	0.0058 (11)	0.0148 (9)	0.0028 (10)
O1	0.0154 (7)	0.0210 (8)	0.0148 (7)	−0.0006 (5)	0.0070 (5)	−0.0011 (6)
O2	0.0128 (7)	0.0214 (8)	0.0151 (7)	−0.0006 (5)	0.0060 (5)	−0.0033 (5)
O3	0.0236 (8)	0.0228 (9)	0.0177 (7)	0.0006 (6)	0.0057 (6)	−0.0018 (6)
O4	0.0282 (8)	0.0212 (8)	0.0175 (7)	−0.0017 (6)	0.0099 (6)	0.0028 (6)
O5	0.0204 (8)	0.0479 (11)	0.0198 (8)	0.0063 (7)	0.0094 (6)	−0.0001 (7)
O6	0.0283 (8)	0.0319 (9)	0.0100 (7)	0.0043 (6)	0.0054 (6)	0.0023 (6)

### Geometric parameters (Å, º)

**Table:**

C1—C2	1.558 (3)	C10—O2	1.449 (2)
C1—C6	1.558 (3)	C10—C11	1.511 (2)
C1—C10	1.568 (2)	C11—C12	1.538 (3)
C1—H1	1.0000	C11—H11A	0.9900
C2—O1	1.454 (2)	C11—H11B	0.9900
C2—C13	1.509 (3)	C12—C13	1.539 (3)
C2—C3	1.536 (3)	C12—H12A	0.9900
C3—C4	1.337 (3)	C12—H12B	0.9900
C3—H3	0.9500	C13—H13A	0.9900
C4—C5	1.525 (3)	C13—H13B	0.9900
C4—H4	0.9500	C14—O3	1.214 (2)
C5—O1	1.453 (2)	C14—O4	1.351 (2)
C5—C6	1.567 (2)	C15—O4	1.448 (2)
C5—H5	1.0000	C15—H15A	0.9800
C6—C7	1.559 (3)	C15—H15B	0.9800
C6—H6	1.0000	C15—H15C	0.9800
C7—O2	1.456 (2)	C16—O5	1.215 (2)
C7—C8	1.534 (2)	C16—O6	1.331 (2)
C7—H7	1.0000	C17—O6	1.461 (2)
C8—C9	1.343 (3)	C17—H17A	0.9800
C8—C14	1.479 (3)	C17—H17B	0.9800
C9—C16	1.493 (2)	C17—H17C	0.9800
C9—C10	1.549 (3)		
			
C2—C1—C6	102.44 (14)	C11—C10—C9	120.61 (15)
C2—C1—C10	112.21 (14)	O2—C10—C1	102.48 (13)
C6—C1—C10	102.16 (13)	C11—C10—C1	114.30 (14)
C2—C1—H1	113.0	C9—C10—C1	104.17 (14)
C6—C1—H1	113.0	C10—C11—C12	111.59 (15)
C10—C1—H1	113.0	C10—C11—H11A	109.3
O1—C2—C13	112.43 (15)	C12—C11—H11A	109.3
O1—C2—C3	100.43 (13)	C10—C11—H11B	109.3
C13—C2—C3	120.53 (16)	C12—C11—H11B	109.3
O1—C2—C1	101.74 (14)	H11A—C11—H11B	108.0
C13—C2—C1	114.14 (15)	C11—C12—C13	112.36 (15)
C3—C2—C1	105.17 (14)	C11—C12—H12A	109.1
C4—C3—C2	105.69 (16)	C13—C12—H12A	109.1
C4—C3—H3	127.2	C11—C12—H12B	109.1
C2—C3—H3	127.2	C13—C12—H12B	109.1
C3—C4—C5	105.73 (17)	H12A—C12—H12B	107.9
C3—C4—H4	127.1	C2—C13—C12	111.82 (16)
C5—C4—H4	127.1	C2—C13—H13A	109.3
O1—C5—C4	101.15 (15)	C12—C13—H13A	109.3
O1—C5—C6	102.15 (13)	C2—C13—H13B	109.3
C4—C5—C6	106.29 (14)	C12—C13—H13B	109.3
O1—C5—H5	115.2	H13A—C13—H13B	107.9
C4—C5—H5	115.2	O3—C14—O4	124.10 (17)
C6—C5—H5	115.2	O3—C14—C8	123.61 (16)
C1—C6—C7	100.74 (13)	O4—C14—C8	112.28 (15)
C1—C6—C5	100.02 (14)	O4—C15—H15A	109.5
C7—C6—C5	116.80 (14)	O4—C15—H15B	109.5
C1—C6—H6	112.6	H15A—C15—H15B	109.5
C7—C6—H6	112.6	O4—C15—H15C	109.5
C5—C6—H6	112.6	H15A—C15—H15C	109.5
O2—C7—C8	100.19 (13)	H15B—C15—H15C	109.5
O2—C7—C6	102.74 (14)	O5—C16—O6	125.93 (17)
C8—C7—C6	105.62 (13)	O5—C16—C9	122.01 (16)
O2—C7—H7	115.5	O6—C16—C9	112.01 (15)
C8—C7—H7	115.5	O6—C17—H17A	109.5
C6—C7—H7	115.5	O6—C17—H17B	109.5
C9—C8—C14	130.17 (16)	H17A—C17—H17B	109.5
C9—C8—C7	106.03 (16)	O6—C17—H17C	109.5
C14—C8—C7	123.00 (15)	H17A—C17—H17C	109.5
C8—C9—C16	130.09 (17)	H17B—C17—H17C	109.5
C8—C9—C10	105.42 (15)	C5—O1—C2	96.38 (13)
C16—C9—C10	123.29 (15)	C10—O2—C7	97.18 (12)
O2—C10—C11	113.16 (14)	C14—O4—C15	115.69 (15)
O2—C10—C9	99.72 (14)	C16—O6—C17	116.06 (15)
			
C6—C1—C2—O1	−33.60 (15)	C16—C9—C10—C1	−96.67 (18)
C10—C1—C2—O1	75.25 (16)	C2—C1—C10—O2	−76.85 (16)
C6—C1—C2—C13	−154.94 (14)	C6—C1—C10—O2	32.17 (16)
C10—C1—C2—C13	−46.10 (19)	C2—C1—C10—C11	45.96 (19)
C6—C1—C2—C3	70.76 (16)	C6—C1—C10—C11	154.98 (14)
C10—C1—C2—C3	179.61 (14)	C2—C1—C10—C9	179.61 (14)
O1—C2—C3—C4	33.34 (19)	C6—C1—C10—C9	−71.37 (16)
C13—C2—C3—C4	157.33 (17)	O2—C10—C11—C12	66.21 (19)
C1—C2—C3—C4	−71.98 (18)	C9—C10—C11—C12	−176.00 (15)
C2—C3—C4—C5	−0.86 (19)	C1—C10—C11—C12	−50.6 (2)
C3—C4—C5—O1	−31.98 (18)	C10—C11—C12—C13	55.1 (2)
C3—C4—C5—C6	74.35 (19)	O1—C2—C13—C12	−64.1 (2)
C2—C1—C6—C7	118.41 (14)	C3—C2—C13—C12	177.84 (15)
C10—C1—C6—C7	2.08 (16)	C1—C2—C13—C12	51.2 (2)
C2—C1—C6—C5	−1.57 (15)	C11—C12—C13—C2	−55.4 (2)
C10—C1—C6—C5	−117.90 (14)	C9—C8—C14—O3	170.32 (18)
O1—C5—C6—C1	36.44 (15)	C7—C8—C14—O3	2.1 (3)
C4—C5—C6—C1	−69.17 (17)	C9—C8—C14—O4	−8.8 (3)
O1—C5—C6—C7	−71.09 (18)	C7—C8—C14—O4	−177.03 (14)
C4—C5—C6—C7	−176.70 (16)	C8—C9—C16—O5	−102.8 (2)
C1—C6—C7—O2	−35.69 (15)	C10—C9—C16—O5	62.9 (3)
C5—C6—C7—O2	71.42 (17)	C8—C9—C16—O6	79.6 (2)
C1—C6—C7—C8	68.87 (17)	C10—C9—C16—O6	−114.78 (19)
C5—C6—C7—C8	175.98 (15)	C4—C5—O1—C2	51.11 (15)
O2—C7—C8—C9	31.95 (17)	C6—C5—O1—C2	−58.46 (15)
C6—C7—C8—C9	−74.48 (17)	C13—C2—O1—C5	179.32 (15)
O2—C7—C8—C14	−157.37 (15)	C3—C2—O1—C5	−51.27 (15)
C6—C7—C8—C14	96.20 (19)	C1—C2—O1—C5	56.79 (14)
C14—C8—C9—C16	−1.3 (3)	C11—C10—O2—C7	−178.39 (14)
C7—C8—C9—C16	168.50 (18)	C9—C10—O2—C7	52.18 (14)
C14—C8—C9—C10	−168.84 (17)	C1—C10—O2—C7	−54.81 (15)
C7—C8—C9—C10	0.92 (17)	C8—C7—O2—C10	−51.89 (15)
C8—C9—C10—O2	−33.65 (17)	C6—C7—O2—C10	56.83 (14)
C16—C9—C10—O2	157.70 (15)	O3—C14—O4—C15	3.6 (3)
C8—C9—C10—C11	−158.04 (16)	C8—C14—O4—C15	−177.27 (15)
C16—C9—C10—C11	33.3 (2)	O5—C16—O6—C17	6.7 (3)
C8—C9—C10—C1	71.98 (16)	C9—C16—O6—C17	−175.75 (15)

### Hydrogen-bond geometry (Å, º)

**Table:**

*D*—H···*A*	*D*—H	H···*A*	*D*···*A*	*D*—H···*A*
C5—H5···O2^i^	1.00	2.58	3.330 (2)	132
C7—H7···O1^i^	1.00	2.52	3.351 (2)	140

Symmetry code: (i) −*x*+1, −*y*+1, −*z*+1.
